# Clinical application of a double-modified sulfated bacterial cellulose scaffold material loaded with FGFR2-modified adipose-derived stem cells in urethral reconstruction

**DOI:** 10.1186/s13287-022-03164-9

**Published:** 2022-09-06

**Authors:** Zhenpeng Zhu, Jiayu Yang, Xing Ji, Zicheng Wang, Chengxiang Dai, Suke Li, Xuesong Li, Yajie Xie, Yudong Zheng, Jian Lin, Liqun Zhou

**Affiliations:** 1 Department of Urology, Peking University First Hospital, Beijing, 100034 China; 2 Institution of Urology, Peking University, Beijing, 100034 China; 3 University of Science and Technology, Beijing, Beijing, 100083 China; 4 Beijing Key Laboratory of Urogenital Diseases (Male) Molecular Diagnosis and Treatment Center, Beijing, 100034 China; 5 Department of Urology, The First Affiliated Hospital of Anhui Medical University, Hefei, 230000 China; 6 Cellular Biomedicine Group Inc. (CBMG), Shanghai, 200234 China

**Keywords:** Urethral reconstruction, Bacterial cellulose, Adipose-derived stem cell, Sulfonated, Angiogenesis

## Abstract

**Background:**

Urethral stricture and reconstruction are one of the thorny difficult problems in the field of urology. The continuous development of tissue engineering and biomaterials has given new therapeutic thinking to this problem. Bacterial cellulose (BC) is an excellent biomaterial due to its accessibility and strong plasticity. Moreover, adipose-derived stem cells (ADSCs) could enhance their wound healing ability through directional modification.

**Methods:**

First, we used physical drilling and sulfonation in this study to make BC more conducive to cell attachment and degradation. We tested the relevant mechanical properties of these materials. After that, we attached Fibroblast Growth Factor Receptor 2 (FGFR2)-modified ADSCs to the material to construct a urethra for tissue engineering. Afterward, we verified this finding in the male New Zealand rabbit model and carried out immunohistochemical and imaging examinations 1 and 3 months after the operation. At the same time, we detected the potential biological function of FGFR2 by bioinformatics and a cytokine chip.

**Results:**

The results show that the composite has excellent repairability and that this ability is correlated with angiogenesis. The new composite in this study provides new insight and therapeutic methods for urethral reconstruction. The preliminary mechanism showed that FGFR2 could promote angiogenesis and tissue repair by promoting the secretion of Vascular Endothelial Growth Factor A (VEGFA) from ADSCs.

**Conclusions:**

Double-modified sulfonated bacterial cellulose scaffolds combined with FGFR2-modified ADSCs provide new sight and treatments for patients with urethral strictures.

**Supplementary Information:**

The online version contains supplementary material available at 10.1186/s13287-022-03164-9.

## Background

Urethral stricture disease (USD) is an abnormal narrowing of the whole urethra with a low cure rate and high recurrence rate [1, 2]. It remains a challenging clinical disease in urology. Patients with USD are often associated with more complications and discomfort, seriously impacting their quality of life [3, 4]. Unfortunately, there is a lack of clinical consensus regarding the best management for USD [5]. Hence, the suitable and efficient treatment of USD remains significant for urologists.

In recent years, many clinical applications have used many techniques to treat urinary pouch strictures, such as lingual and buccal mucosal patches [6, 7]. However, the limited availability of autologous tissue and the associated complications limit its application. Of interest, the rise of tissue engineering and regenerative medicine technologies has facilitated the development of new biomaterials, which contribute to patients with USD [8]. BC is an excellent nanofibrous material for tissue engineering produced by bacterial fermentation [9]. BC is widely used in tissue repair due to its good mechanical properties and biocompatibility, such as nerve, bone, and urethral tissue [10, 11]. However, BC alone is not degradable in the human body, and it is necessary to modify it.

On the other hand, seed cells and corresponding cytokines also play essential roles in tissue repair. ADSCs are widely used in tissue repair because they are easily accessible and can secrete various cytokines [12, 13]. Targeted modification of ADSCs can promote their secretion of more cytokines and thus stimulate tissue repair. As a protein that broadly influences the FGF signaling pathway, FGFR2 is closely linked to the development and repair effect of the urinary tract [14, 15]. Targeted modification of ADSCs overexpressing FGFR2 may contribute to their secretory function and repair effects.

This study first perforated and sulfated the bacterial cellulose material to make it biocompatible and degradable. Meanwhile, we overexpressed FGFR2 in ADSCs and found that it secreted more pro-angiogenic factors and affected the proliferation and migration ability of ADSCs. The tissue-engineered urethra was further constructed and used for urethral defects in New Zealand rabbits. Afterward, postoperative histological and imaging observations were made. The postoperative results of this tissue-engineered urethra were close to those of a normal urethra, providing a new idea and method for the clinical treatment of USD.

## Methods

### Animal model processing

Eighteen male New Zealand rabbits (4–5 months old; weight 2.5–3.0 kg) were purchased from Fang Yuan Yuan Co., Ltd, Beijing, China. (S.C.X.K. [Jing] 2020–0001). The rabbits were randomly divided into cut-and-suture, FGFR2 Ctrl, and FGFR2 OE groups, with six rabbits in each group. The experiment was approved by the ethics committee (J202064), and the rabbits were housed in individual cages at Peking University First Hospital (SYXK [Jing] 2019-0009). All procedures comply with the ARRIVE guidelines and in accordance with the U.K. Animals (Scientific Procedures) Act, 1986 and associated guidelines.

### Materials processing

In this study, bacterial cellulose was purchased from Hainan Yida Food Co., Ltd. The BC was cut into 2 cm diameter disks and stored at 4 °C. Afterward, the treated BC disk was immersed in different concentrations of sodium periodate solution and soaked for 24 h and 48 h away from light to complete the dehydration of the BC C2 and C3 positions [16, 17]. Then, the BC disk was transferred to a 5 wt% sodium bisulfite (NaHSO3) solution. The reaction was carried out at 50 °C in a water bath for 5 h to complete the sulfonation modification process of BC. Finally, the samples were repeatedly washed with deionized water. AutoCAD software was used to draw standard diagrams of micron-sized pores, and a CO_2_ laser cutter was used to drill to obtain a physical modification. Finally, we constructed the double-modified BC (DMBC).

### Material property testing

The characteristic morphology and chemical structure were detected using a Bruker Tensor II Fourier infrared spectrometer (Brooke (Beijing) Technology Co., Ltd. (Bruker)). Furthermore, energy-dispersive X-ray spectrometry (EDS) was used to observe the distribution of C, O, N, and S elements on the surface of each material group.

After proving the construction of this material again, we tested it accordingly. The static contact angle was measured using an optical contact angle meter (OCA20, Dataphysics Inc.). Then, a mechanical property test was performed according to the ASTMD 628-2003 standard. The stress–strain curve, tensile strength, and elongation at break were measured at a 2 mm/s test speed. Each group was measured three times, and the mean value was taken.

In the degradation experiment, the samples BC and SMBC to be tested were cut into shapes of similar size, placed in a centrifuge tube containing 10 ml of PBS buffer, and placed in a shaker to measure the degradation performance of the samples. The temperature of the shaker was set to 37 °C, the rotating speed was 100 r/min, in order to simulate the human in vivo environment. The degraded samples were taken out at a specific time, dried and weighed, and the mass loss ratio of each sample was calculated.

### Application of ADSCs to SMBC

The SMBC was cut into 2 cm diameter disks and stored at 4 °C. Subsequently, we digested and counted ADSCs that grew to 70–80% and added them to the scaffold material at a concentration of 1 × 10^6^ cell/ml [18, 19]. The medium was changed every 2–3 days for tissue-engineered urethra and the composite was used as a graft for urethral defect repair after 7 days.

### Cell culture and transfection

Shanghai's cellular Biomedicine Group Inc. CBMG kindly provided the ADSC cells and corresponding culture technology. The medium was changed every 2 or 3 days, and the cells were passaged at 70–80% confluence. For lentivirus packaging, we used the three plasmid systems GV492-GFP-puro-FGFR2, psPAX2, and pMD2. G. Then, we used P3-generation ADSCs for lentiviral transfection, resulting in FGFR2 overexpression. Subsequently, we observed transfection efficiency by green fluorescence and performed cell screening by flow cytometry (BD FACSMelody).

### Cellular functional assays

The cell viability (proliferation) of each experimental group was determined using the Cell Counting Kit-8 (CCK8) colorimetric assay (KeyGEN Biotech) according to the manufacturer's instructions. Furthermore, transwell (8 μm) migration and invasion assays were performed using the Costar Transwell system (3422; Corning). According to the manufacturer's protocol, Wayne Biotechnologies (Shanghai) Inc. performed the cytokine array experiment.

### RNA sequencing and functional annotation

We performed RNA sequencing on FGFR2-overexpressing and normal Ctrl ADSC cell lines to assess the underlying molecular biological mechanisms. First, differentially expressed genes between the two groups were screened using the Deseq2 package [20]. Then, Kyoto Encyclopedia of Genes and Genomes (KEGG) and Gene Ontology (GO) pathway enrichment analyses were performed using the ClusterProfiler package to identify potential biological processes. All bioinformatics analysis steps were performed based on R (version 4.0.3) [21].

### Model construction and postoperative evaluation

The prepared materials were cut into disks with a diameter of 2.2 cm and sterilized by 60CO irradiation. Our material was then moistened with saline, and cells were loaded onto the material to construct the tissue-engineered urethra. By lentiviral transfection, we constructed ADSCs cell lines with FGFR2 overexpression, which were loaded onto SMBC. The FGFR2-Ctrl and FGFR2-OE groups were constructed separately, while the cut-and-sewn ones were used as negative control groups. Subsequently, we randomly divided 18 healthy male New Zealand rabbits into 3 groups (n = 6): the FGFR2 OE group, the FGFR2 Ctrl group, and the negative control group. The surgeons are all specialist urologists, and the Ethics Committee approved all experimental animal procedures of Peking University First Hospital.

The New Zealand rabbit was fixed on the manipulator and anesthetized with pentobarbital sodium along the marginal ear vein. Subsequently, a layer-by-layer separation is made to the urethra, and a partial anterior urethral defect of approximately 1.5 cm is incised. Postoperatively, an 8F catheter was routinely left in place, and the head was immobilized with an Elizabethan ring for one week. Meanwhile, the antibiotics were routinely administered intramuscularly for 3 days. Urethrography and ureteroscopy were performed to observe the general condition of the urethra. Hematoxylin–eosin (HE), Masson, and immunohistochemistry-paraffin (IHC-P) were used to determine the histological structure of the repair site.

### Statistical analysis

The data in the figures are presented as the mean and standard error of the mean for each group. Individual comparisons were made between groups using two-tailed Student's *t* tests. One-way ANOVAs analyses were used to screen out the difference in multiple groups. *P* values < 0.05 were considered significant.

## Results

### The processes and internal microstructures of double-modified sulfonated bacterial cellulose

**Fig. 1:**
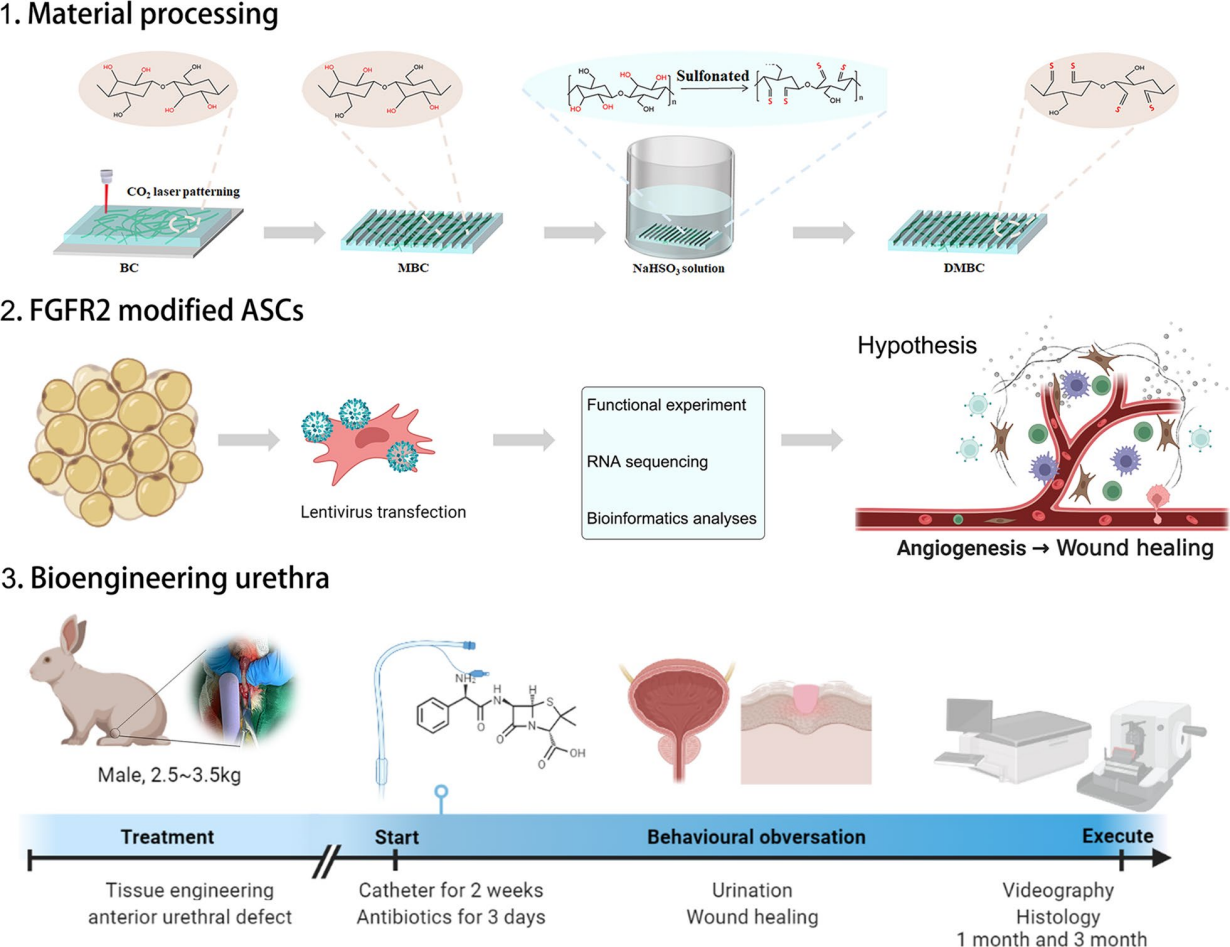
Flowchart of the whole experimental processes

Figure 1 shows the whole process of this study. First, we made the laser hole to physically modify BC using the strip micro holes. Previous studies have shown that targeted modification of BC can enhance its biological activity and repairability. Hence, we sulfonated the hydroxyl groups of modified BC (MBC) at the C2 and C3 positions to obtain high bioaffinity and degradable sulfonated MBC (SMBC) materials. Then, we overexpressed FGFR2 in ADSCs by lentiviral infection and evaluated its potential function by cell function, RNA-seq, and cytokine microarrays. Third, we constructed a tissue-engineered urethra and evaluated it with a New Zealand rabbit model. All the processes were drawn using the BioRender.com.

**Fig. 2:**
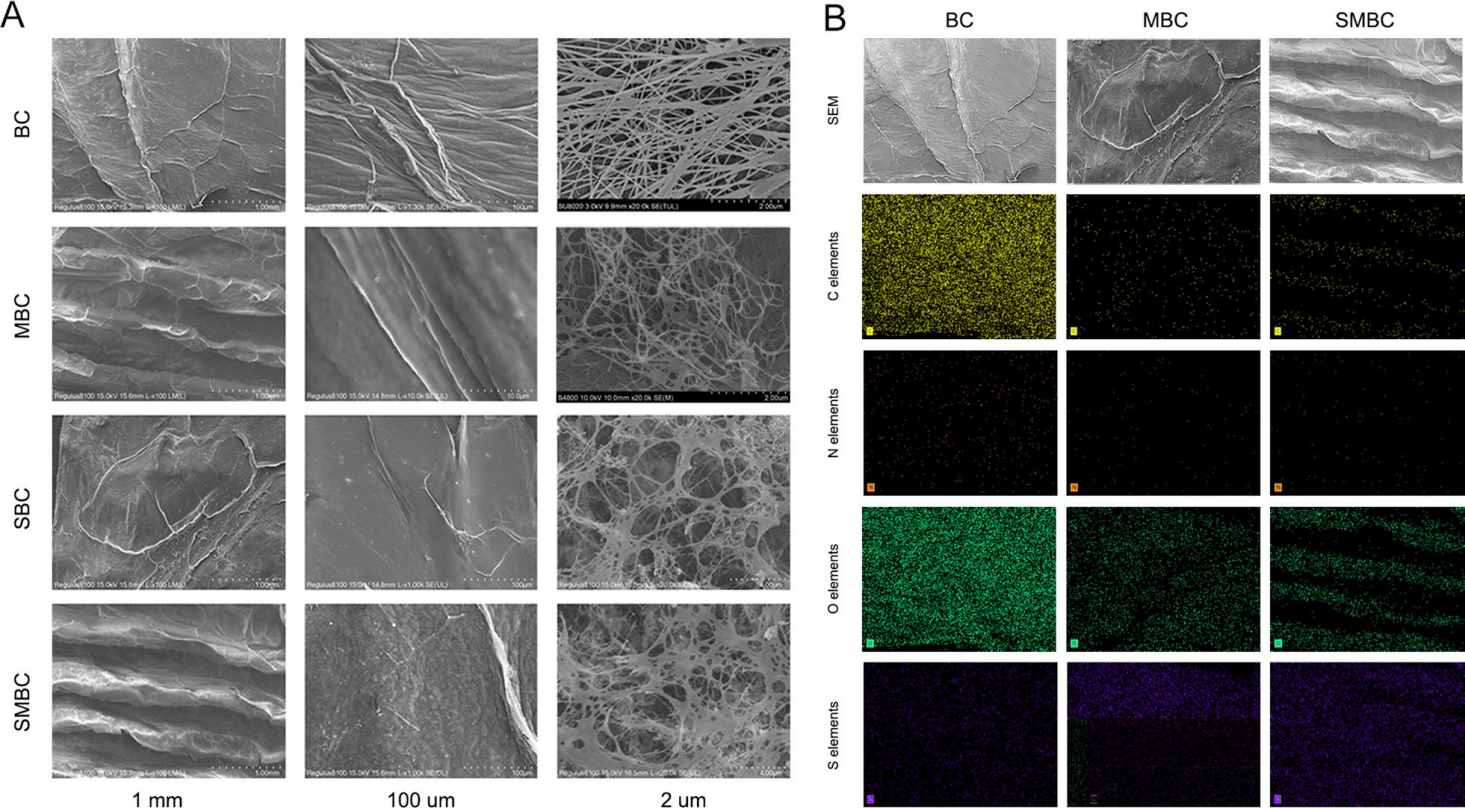
**A** Microstructure of BC, MBC, SBC, and SMBC; **B** surface scan images of BC, MBC, SBC, and SMBC

Parts a–b of Fig. 2 show the internal microstructures of BC, MBC, SBC, and SMBC. As shown in Fig. 2A, the surface of the unmodified BC shows a flat mesh structure without micropores and other special structures. When performing a laser punch to construct the MBC, the materials showed parallel and regular striated structures, ranging from 200 to 300 µm in size. The MBC can provide for cell attachment and growth. Furthermore, we sulfonated the BC material, and it is obvious that the nanofibers of SBC are more irregularly arranged and have a rougher surface. Subsequently, we double-modified the BC by sulfonation treatment and laser perforation, and the DMBC had denser grooves and a rougher surface. Some nanofibers were intertwined. The microstructure of DMBC provides a good basis for cell adhesion.

After successfully constructing the double-modified DMBC and observing its internal microstructure, we performed surface scans of BC, SBC, and DMBC by energy-dispersive X-ray spectrometry (EDS). As shown in Fig. 2B, we analyzed the internal elements of the material and found that the DMBC material contained more S elements and fewer C, N, and O elements. EDS indirectly proved that DMBC facilitated cell adhesion, and the effects of S-containing materials have been well studied in several studies [22, 23].

### Mechanical properties and biocompatibility of double-modified sulfonated bacterial cellulose

**Fig. 3:**
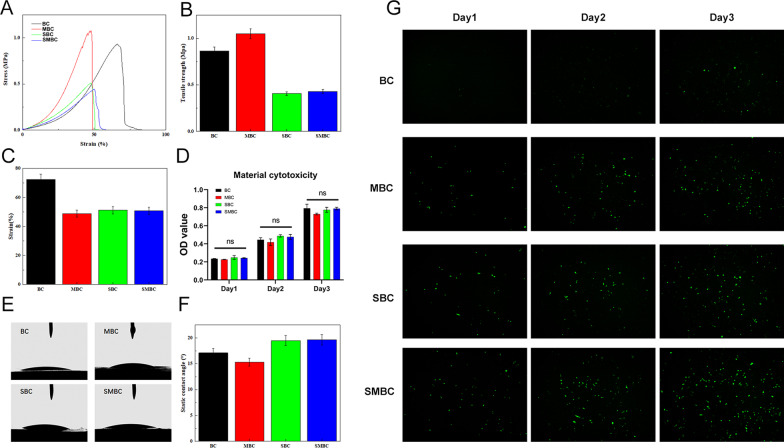
**A** Stress − strain curves, **B** tensile strength, and **C** elongation at break of BC, MBC, SBC, and SMBC; **D** biocompatibility of BC, MBC, SBC, and SMBC, “ns”: no significance; **E**, **F** Contact angles of BC, MBC, SBC, and SMBC; (g) GFP fluorescence of BC, MBC, SBC, SMBC; the scale bar is 100 μm

The reduction in carbon compared to BC or SBC makes us wonder if it causes a weakening of the mechanical properties [24]. The tensile mechanical property is one of the very important indicators in urethral reconstruction [25]. The maintenance of tensile mechanical properties helps prevent the collapse of the nascent urethra and the creeping in of epithelial cells. As shown in Fig. 3A–C, we drew the tensile stress–strain curves of BC, MBC, SBC, and DMBC using a mechanical testing machine. The results showed that the tensile strength of BC is 0.86 ± 0.043 MPa, and that of MBC is 1.05 ± 0.052 MPa. The tensile strength of MBC is close to that of BC, which indicates that the physical modification of BC does not significantly change the tensile strength of BC. The tensile strength of SBC is 0.41 ± 0.020 MPa, which is obviously lower than that of BC. The tensile strength of SMBC is 0.43 ± 0.022 MPa, which is similar to that of SBC, but significantly different from that of MBC. This indicates that the decrease in the tensile strength of SMBC is mainly due to the chemical modification of BC, which has little relationship with physical modification. The elongation at break of BC, MBC, SBC, and SMBC is 72.31%, 48.77%, 51.15% and 50.79%, respectively, which indicates that the physical and chemical modification of BC will make the elongation at break smaller. In conclusion, although the mechanical properties of SMBC materials decreased to a certain extent compared with those of BC, they still met the requirements of mechanical properties.

Furthermore, we explored different materials and whether S-containing elements can be toxic to cells. Figure 3D shows that material extracts do not produce toxic effects on cells and do not inhibit the proliferative state of cells. Figure 3e and f shows the hydrophilicity and hydrophobicity of BC, BC, MBC, SBC, and SMBC. BC is extremely hydrophilic, with a contact angle of 17.1° ± 0.86°. The contact angle of MBC material is 15.3° ± 0.77°, which is slightly lower than that of BC, while the contact angles of SBC and SMBC are increased to a certain extent, which are 19.5° ± 0.97° and 19.7° ± 0.98°, respectively. This is because in the process of oxidation and sulfonation, part of the hydroxyl groups of BC are converted into sulfonic acid groups, and the hydrophilicity of the sulfonic acid group is slightly stronger than that of the hydroxyl groups. Finally, after comparing a series of material properties, we loaded ADSCs onto different materials. As shown in Fig. 3G, we found that the SMBC group of materials loaded with more ADSCs, while the SBC and MBC groups did not show significant differences.

### Overexpression of FGFR2 enhances the wound healing capability of ADSCs

**Fig. 4:**
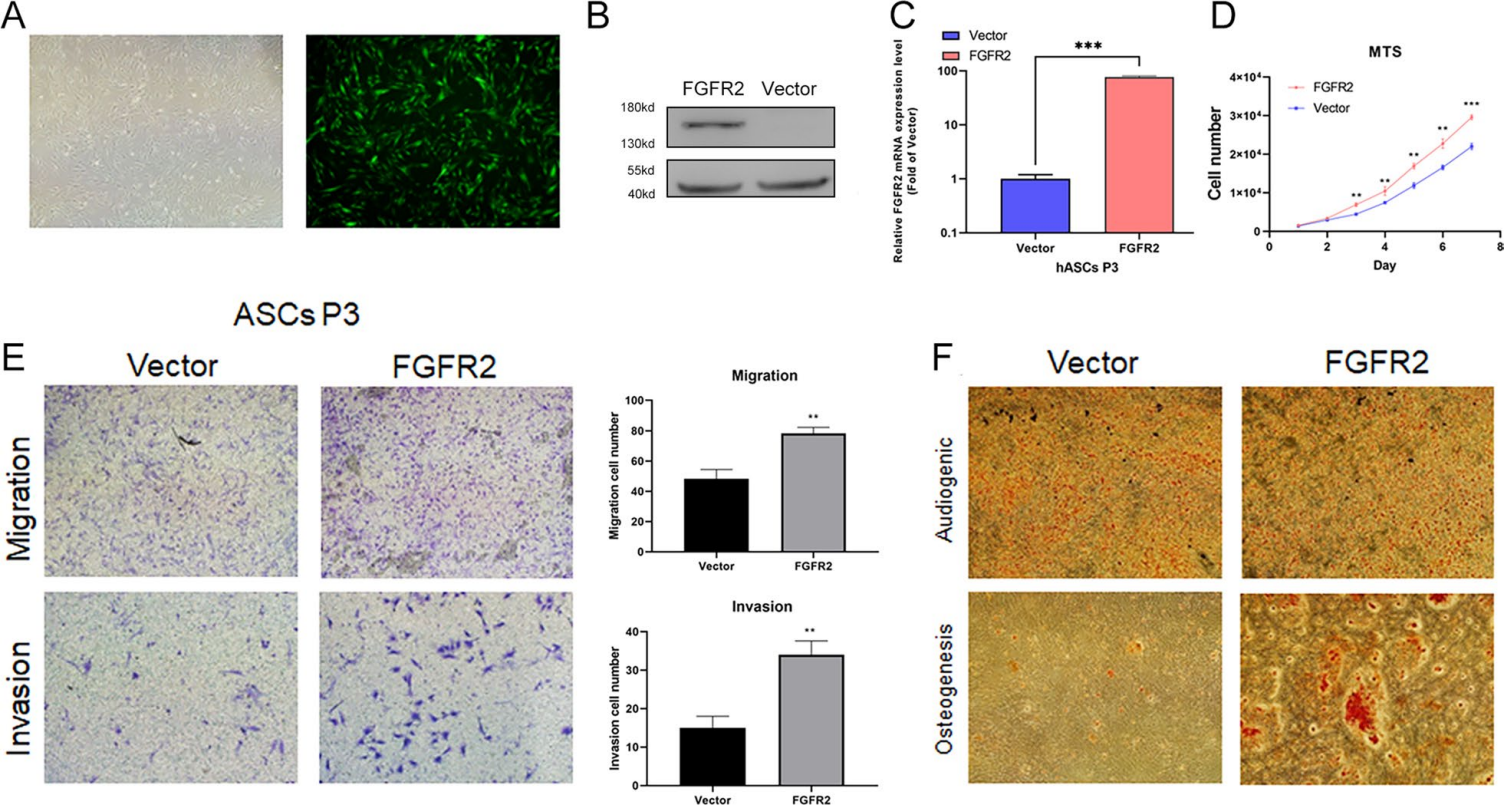
**A** ADSCs cells under white light and fluorescence; **B**, **C** Partial size of protein and mRNA expression levels of overexpressed FGFR2; **D**, **E** proliferation, migration and invasion ability of FGFR2 ctrl and OE ADSCs; **F** Osteogenic and adipogenic differentiation capacity of FGFR2 ctrl and OE ADSCs. “**”, < 0.01; “***”, < 0.001

After exploring the mechanical properties of the building materials, we further modified the seed cells using lentivirus. As shown in Fig. 4A–C, we constructed ADSC cell lines of FGFR2 Ctrl and FGFR2 OE and verified their protein and mRNA expression levels by western blot (Fig. 4B) and qPCR (Fig. 4C). We then tested whether FGFR2 would have a corresponding effect on ADSCs themselves. As shown in Fig. 4D, FGFR2 overexpression significantly enhanced the proliferative capacity of ADSCs (from day 3 onward). Subsequently, Fig. 4E shows that FGFR2 can enhance the invasion and migration of ADSCs, which may contribute to their colonization of wound sites from scaffolds. Finally, the differentiation experiments in Fig. 4F show that FGFR2 enhanced the osteogenic capacity of ADSCs without greatly affecting lipogenic capacity, which is consistent with previous studies.

### Bioinformatics analysis suggests that FGFR2 can promote the secretion capacity of ADSCs

**Fig. 5:**
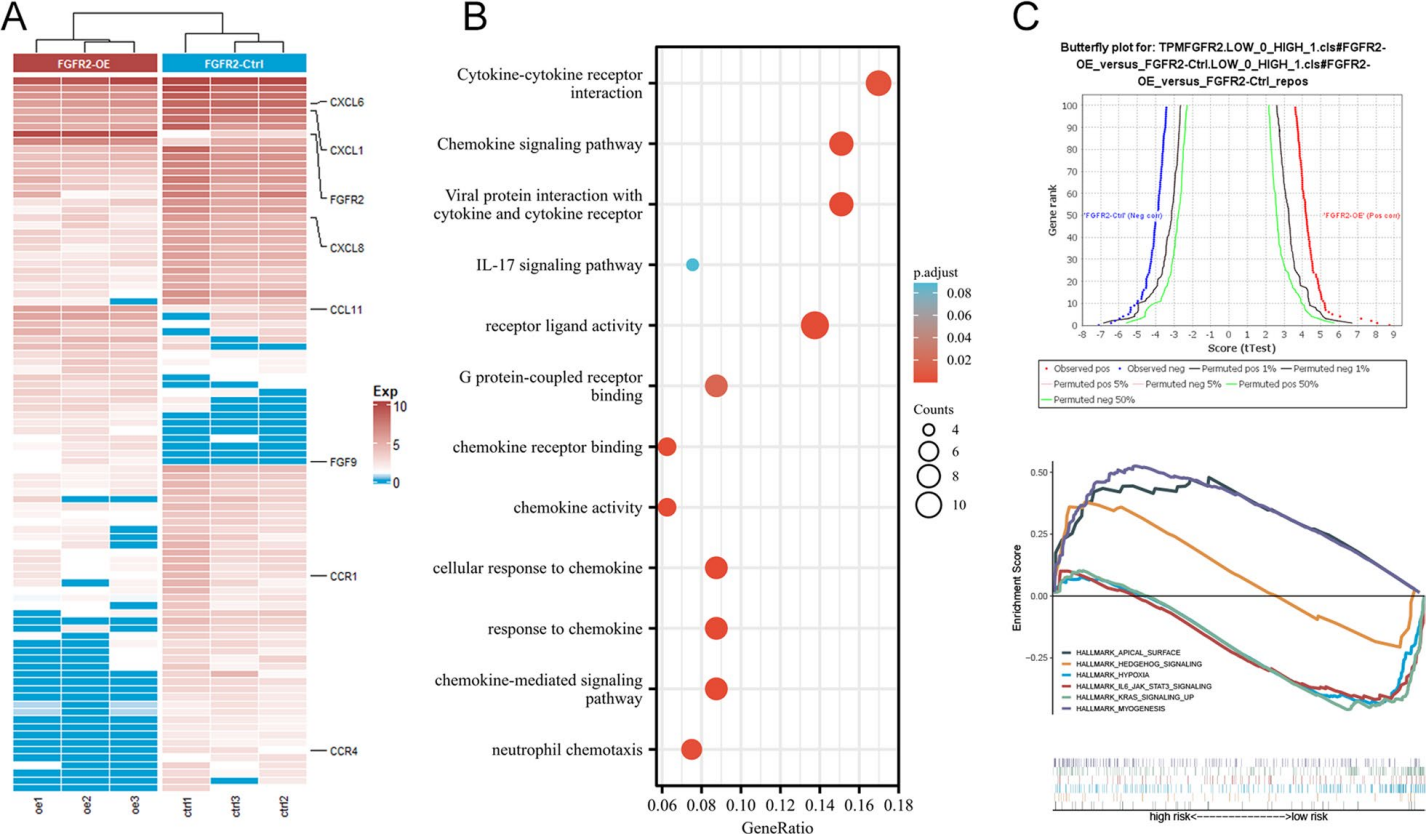
**A** Differential gene heatmap between two groups; **B** KEGG enrichment analyses of the differential genes; **C** GSEA enrichment analyses of the two groups

Afterward, we explored the potential molecular biological mechanisms by which FGFR2 affects the repair capacity of ADSCs. Previous studies suggest that ADSCs affect tissue repair mainly in a paracrine manner, which is the main mechanism by which some heterozygous ADSCs exert their functions [26, 27]. We detected the relevant mRNA alterations in FGFR2 ctrl and OE group ADSCs by RNAsequencing technology, as shown in Fig. 5A. We found significant alterations in some cytokines, such as CXCL8 and CXCL1. Meanwhile, enrichment by KEGG analyses (Fig. 5B) showed that the differentially expressed genes were mainly enriched in cytokine-related pathways. Figure 5C indicates that FGFR2 may be enriched in the hedgehog pathway, hypoxia pathway, and inflammation-related pathways. This suggests that FGFR2 might regulate the secretion, stemness, and inflammatory factor secretion of ADSCs [28].

### FGFR2 can promote ADSCs to secrete VEGFA to promote angiogenesis

**Fig. 6:**
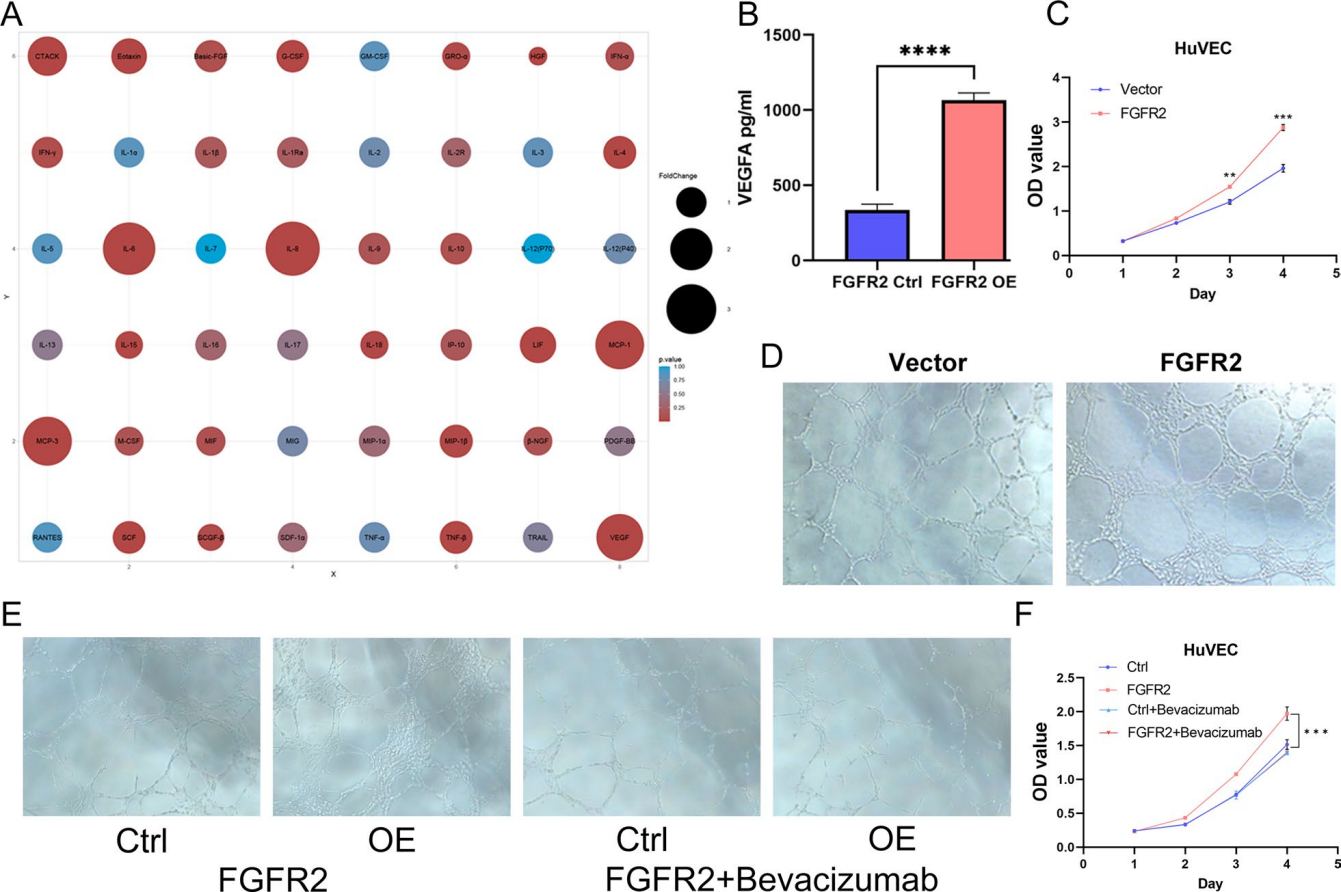
**A** Differential cytokines dot plot between two groups; **B** Elisa analyses of the VEGFA between two groups; **C** Proliferation ability of HuVECs in different cell supernatants treated; **D** Angiogenic capacity of HuVECs under co-culture conditions (×200). **E** Angiogenic capacity of HuVECs under co-culture conditions and Bevacizumab (500 nM) (×200). **F** Proliferation ability of HuVECs in different cell supernatants treated and Bevacizumab (500 nM)

Since there may be an association between FGFR2 and the secretory function of ADSCs, we examined the changes in common secretory proteins in the supernatants of ADSC cells in the FGFR2 ctrl and OE groups by cytokine microarrays. Figure 6A shows the changed cytokines, the most significant of which are VEGFA, IL-6, IL-8, etc. VEGFA is highly conserved among species, and previous studies suggest a role for ADSCs in angiogenesis [29, 30]. We verified the expression level of VEGFA in the cell supernatant, and indeed, overexpression of FGFR2 significantly upregulated the expression level of VEGFA, as shown in Fig. 6B. We then examined the effects of FGFR2 ctrl and OE group supernatants on the proliferation of HuVECs under in vitro conditions, as well as the effects of FGFR2 on the angiogenic capacity of HuVECs by coculture. Figure 6C and D shows that FGFR2 enhances the proangiogenic capacity of ADSCs, which may be dependent on VEGFA. Subsequently, we explored whether FGFR2 exerted its corresponding function through VEGFA. We obtained an antibody to VEGFA, that was Bevacizumab. The corresponding conditioned medium was obtained by adding Bevacizumab to the supernatant of ADSCs. It was then used to assay the proliferation and proangiogenic capacity on HuVECs cells. As shown in Fig. 6E and F, the promotion of FGFR2 on the proliferation and proangiogenic capacity of HuVECs was lost when Bevacizumab was added.

### In vivo experiments reveal the effectiveness of this tissue-engineered urethra

**Fig. 7:**
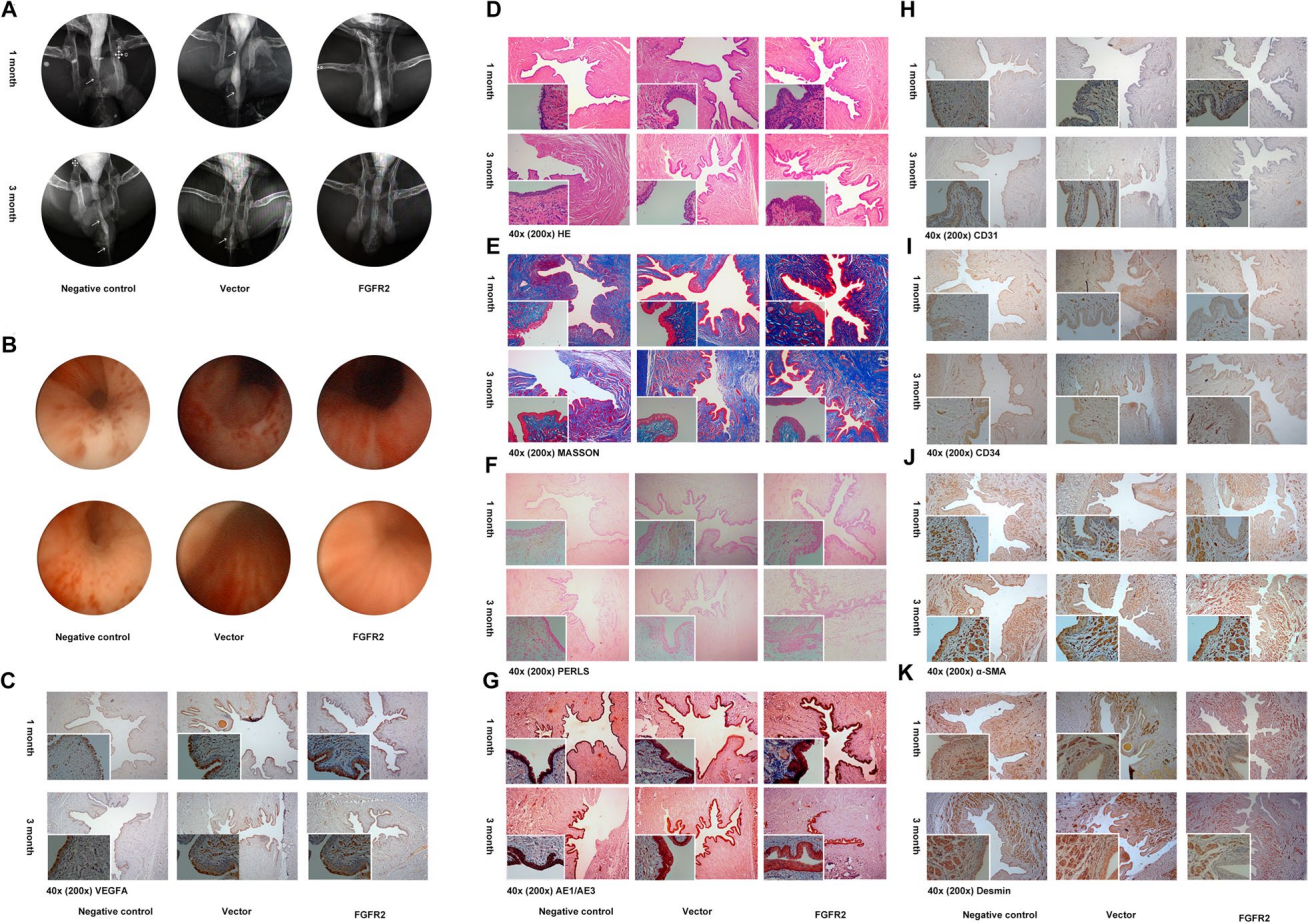
**A**, **B** Urography and urethroscopy of Negative controls, FGFR2 ctrl and FGFR2 OE groups at 1 month and 3 months after surgery; **C** VEGFA; **D** HE; **E** MASSON; **F** Prussian blue; **G** AE1/AE3; **H** CD31; **I** CD34; **J** alpha-SMA; **K** Desmin of Negative controls, FGFR2 ctrl and FGFR2 OE groups at 1 month and 3 months after surgery

We first examined the in vitro degradation of BC material and SMBC material, where significant degradation of SMBC material could be seen at 30 days, while BC material was intact (Additional file 1: Fig. S1A). The same changes were also shown under electron microscopic microstructure at 0 and 7 days (Additional file 1: Fig. S1B). After developing scaffold materials and seed cells, we lectured on FGFR2-modified ADSCs loaded onto SMBC and performed in vivo trials in a New Zealand rabbit urethral defect model. Figure 7A and B shows the urography and urethroscopy of the negative control group, the FGFR2 ctrl group, and the FGFR2 OE group at 1 and 3 months postoperatively. It can be seen that the FGFR2 OE group had better postoperative results, with urethral continuity and integrity maintained and close to normal urethral tissue. As shown in Table 1, urethral leakage or severe urethral stricture resulted in the death of one New Zealand rabbit in each of the negative control and FGFR2 ctrl groups at 3 months. We then evaluated the repaired tissue by IHC-P and a series of stainings, such as HE and Masson staining. As shown in Fig. 7C, the staining depth of VEGFA was significantly stronger in the FGFR2 OE group than in the other two groups and showed a more complete vascular morphology. Figure 7D shows that the FGFR2 OE group had a more complete and dense epithelial covering and showed good restorative properties at 1 month, while Fig. 7E shows that the FGFR2 OE group had a more regular arrangement of fibers and showed less possibility of scar formation in the repaired tissue. Figure 7F shows that the FGFR2 OE group showed less inflammatory infiltration and bleeding at 1 and 3 months postoperatively, which could be closely related to better repair results. Additionally, Fig. 7G is similar to Fig. 7D and shows an increased number of epithelial layers and a more complete epithelial recovery in the FGFR2 OE group after surgery. Figure 7H and I shows the number of neovascularization and tube sizes by CD31 and CD34, and the results suggest that neovascularization was greater in the FGFR2 OE group at 1 month postoperatively, while it was not as obvious at 3 months. Finally, Fig. 7J and K shows the recovery of the smooth muscle layer after surgery by desmin and α-SMA, which is important for urethral repair. The smooth muscle layer in the FGFR2 OE group had better continuity and a more regular structure.

**Table 1:** Detailed follow-ups of rabbits between different groups

	Negative controls						FGFR2 Ctrl						FGFR2 OE					
Animal ID	1	2	3	4	5	6	7	8	9	10	11	12	13	14	15	16	17	18
State at the end point	Alive	Alive	Alive	Alive	Dead	Alive	Alive	Alive	Alive	Alive	Alive	Dead	Alive	Alive	Alive	Alive	Alive	Alive
Endpoint	1 month	1 month	1 month	3 month	3 month	3 month	1 month	1 month	1 month	3 month	3 month	3 month	1 month	1 month	1 month	3 month	3 month	3 month
Grossly:																		
*Fistula*	+	−	+	+ + +	+ + +	+	+ +	+	+ +	−	−	+ + +	−	+	−	+	−	−
*Stone*	−	−	−	+	−	+	−	−	−	−	−	+	−	−	−	−	−	−
Histology																		
*Urothelium*	+	+	+	+ +	+	+ +	+ +	+	+ +	+ +	+ +	+	+ + +	+ + +	+ +	+ + +	+ + +	+ + +
*SMC*	+ +	+	+	+ +	+ +	+ +	+ +	+	+ +	+ +	+ +	+	+ +	+ + +	+ +	+ + +	+ + +	+ +
*Scarring*	+ + +	+ +	+	+	+ + +	+	+	+	+ + +	+	−	+ +	−	+	−	−	−	+
*Angiogenesis*	+	+	+ +	+	+	+	+	+ +	+	+ +	+	+ +	+ +	−	+ +	+ + +	+ +	+ +
Other complications	−	−	−	−	−	−	−	−	−	−	−	−	−	−	−	−	−	−

0–3 + , "−" Negative; "+" Weak; "++" Moderate; "+++" Strong

## Discussion

USD, especially the long-segment complex USD, remains a thorny problem in the field of urology due to its recurrence and various complications [1, 2]. The materials currently used in clinical practice, whether autologous or synthetic, have certain drawbacks. With the continuous advancement of biomaterials and cell modification techniques, tissue engineering could reduce the financial burden on patients and improve their treatment outcomes due to its advantages [31, 32]. However, the construction of a suitable tissue-engineered urethra still requires exploration. The building of bioengineered tissue often needs to be considered for both the suitable biomaterials and seed cells. Then, finding the right combination was identified to better repair the corresponding tissue.

Bacterial cellulose is a novel bioscaffold material that is cost-effective and readily available [33, 34]. Due to the nondegradable nature of BC, directional modification of the BC material to make it more compatible with the application of the reconstruction site is of paramount importance. Previous studies have enhanced the degradability of BC by oxidizing it. We have also improved biocompatibility and degradability by oxidizing and perforating it and binding it to soy protein for urethral repair [35]. In the present study, we sulfonated and perforated the B.C Compared to BC or single modified BC, DMBC retains its mechanical properties better, while its biocompatibility and degradability are significantly enhanced. This gives us a glimpse of the potential value of its clinical application.

Furthermore, ADSCs are excellent seed cells that are easy to obtain and negative for tumorigenesis tests [36]. Previous studies have shown that the targeted modification of ADSCs could enhance their tissue repair capacity [37, 38]. The literature previously reported that overexpression of FGFR2 can promote wound healing, but the exact mechanism remains to be screened [39, 40]. Marissa et al. demonstrated that FGFR2 plays an important role in the normal development of the external genitalia and that knockdown of FGFR2 in endodermal cells resulted in hypospadias and inhibition of urethral epithelial maturation, while knockdown of FGFR2 in ectodermal cells resulted in severe hypospadias and absence of the ventral foreskin [15]. Hence, in this study, we hypothesized that overexpression of FGFR2 might promote the tissue repair capacity of ADSCs. Moreover, RNA sequencing analyses were performed to explore the potential biological functions of FGFR2, using the GO, KEGG and GSEA enrichment analyses. We found that FGFR2 overexpression was enriched in some of the cytokine secretion-related pathways. In subsequent experiments, we also found that FGFR2 does have a promotive effect on angiogenesis, which may be an important factor in its ability to enhance tissue repair. Ultimately, we validated the above experimental results in a New Zealand rabbit urethral defect model.

In this study, we developed a new tissue-engineered urethra and obtained satisfactory outcomes in both postoperative histological and imaging tests. However, there are certain limitations in this study that need to be explored in subsequent experiments. First, the therapeutic effect of allogeneic ADSCs was assessed in this study based on their ability of pro-secretory factors. Whether FGFR2 can promote the differentiation of ADSCs remains to be investigated. Second, we used lentiviral-transfected ADSCs in this study and it might be of more clinical value to use exosomes or to confirm elevated levels of VEGFA expression in exosomes. Finally, the biomaterial can be further improved in the future by 3D bioprinting or by slowing down the release of certain factors (e.g., VEGFA).

## Conclusions

At present, there is still a lack of effective treatment for USD, especially for long and complex USD. Former studies have used autologous tissue as repair material, but the methods are limited by the amount and complications of the material. In this study, we first obtained the double-modified bacterial cellulose by laser perforation and sulfonation treatment to enhance its cytocompatibility and degradability. At the same time, mechanical properties and cytotoxicity were not significantly altered. We then constructed ADSCs overexpressing FGFR2 and examined the effect of FGFR2 on the secretory capacity of ADSCs by RNA-seq and cytokine microarray. These results tentatively suggest that FGFR2 can enhance the proangiogenic capacity of ADSCs. We subsequently constructed SMBC scaffold material loaded with FGFR2 modification and confirmed the effectiveness of this tissue-engineered urethra in animal models. This study provides new ideas and approaches for the clinical treatment of urethral strictures and reconstruction.

### Supplementary Information

**Additional file 1**. **Figure S1**. (A) In vitro degradation of BC and SMBC materials at 0 and 30 days. (B) Scanning electron microscopy microstructure of BC and SMBC materials at 0 and 7 days of in vitro degradation (100 µm)

## Data Availability

All data generated or analyzed during this study are included in this published article.

## Abbreviations

ADSCs: Adipose-derived stem cells
BC: Bacterial cellulose
USD: Urethral stricture disease
FGFR2: Fibroblast Growth Factor Receptor 2
VEGFA: Vascular Endothelial Growth Factor A
MBC: Modified BC
SMBC: Sulfonated MBC
EDS: Energy-dispersive X-ray spectrometry
KEGG: Kyoto Encyclopedia of Genes and Genomes
GO: Gene Ontology
qPCR: Quantitative polymerase chain reaction
OE: Overexpressed
