# Correction: Clinical application of a double-modified sulfated bacterial cellulose scaffold material loaded with FGFR2-modified adipose-derived stem cells in urethral reconstruction

**DOI:** 10.1186/s13287-023-03541-y

**Published:** 2024-02-27

**Authors:** Zhenpeng Zhu, Jiayu Yang, Xing Ji, Zicheng Wang, Chengxiang Dai, Suke Li, Xuesong Li, Yajie Xie, Yudong Zheng, Jian Lin, Liqun Zhou

**Affiliations:** 1 Department of Urology, Peking University First Hospital, Beijing, 100034 China; 2 Institution of Urology, Peking University, Beijing, 100034 China; 3 University of Science and Technology Beijing, Beijing, 100083 China; 4 Beijing Key Laboratory of Urogenital Diseases (Male) Molecular Diagnosis and Treatment Center, Beijing, 100034 China; 5 Department of Urology, The First Affiliated Hospital of Anhui Medical University, Hefei, 230000 China; 6 Cellular Biomedicine Group Inc. (CBMG), Shanghai, 200234 China

**Correction to: Stem Cell Research and Therapy (2022) 13:463** 10.1186/s13287-022-03164-9

Following the publication of the original article [1], the authors have identified some errors in the Figs. 2, 4, 5, 7 and Additional file 1: Fig. S1. The correction are as follows:

**Fig. 2:**
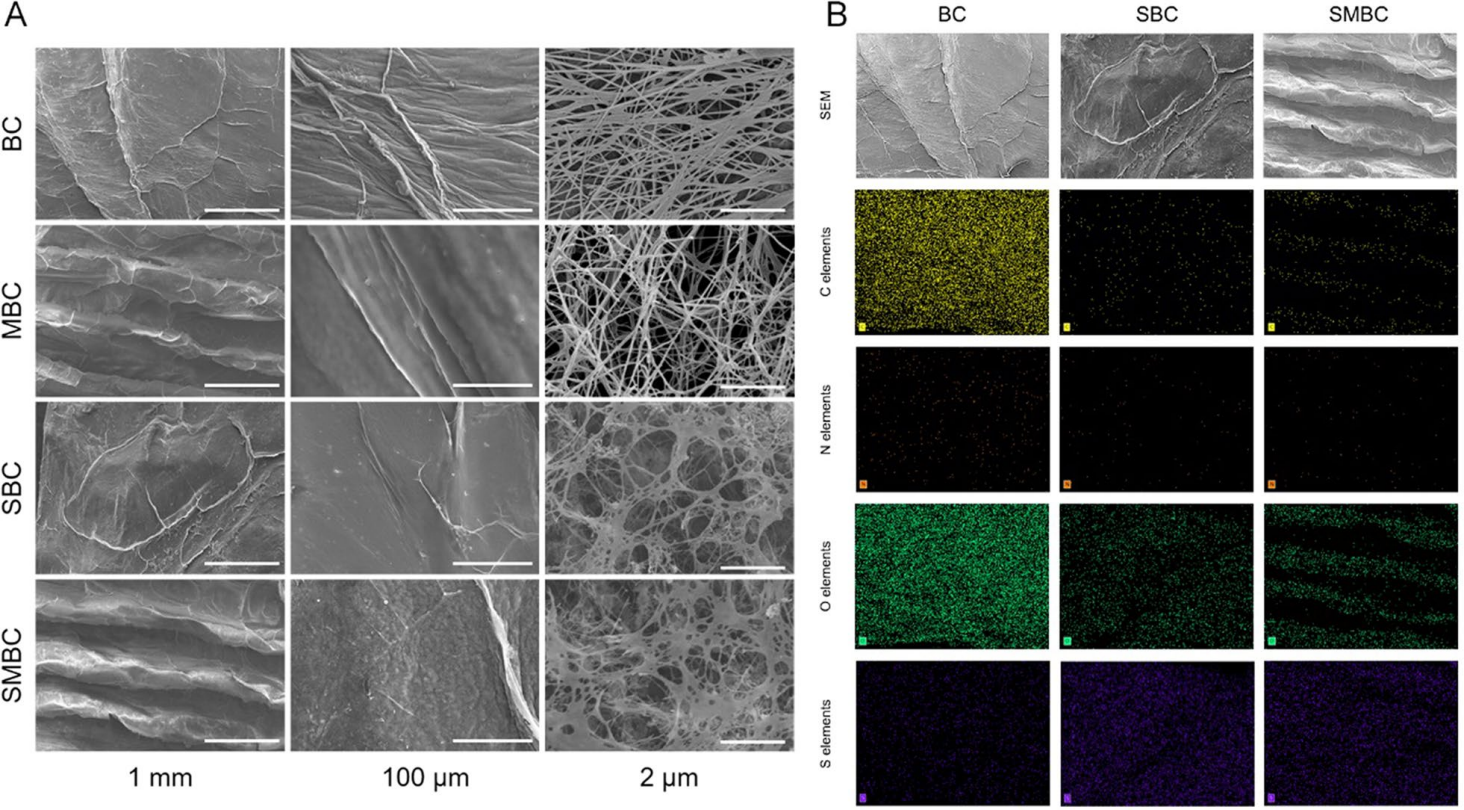
**A** Microstructure of BC, MBC, SBC, and SMBC; **B** surface scan images of BC, SBC, and SMBC

**Fig. 4:**
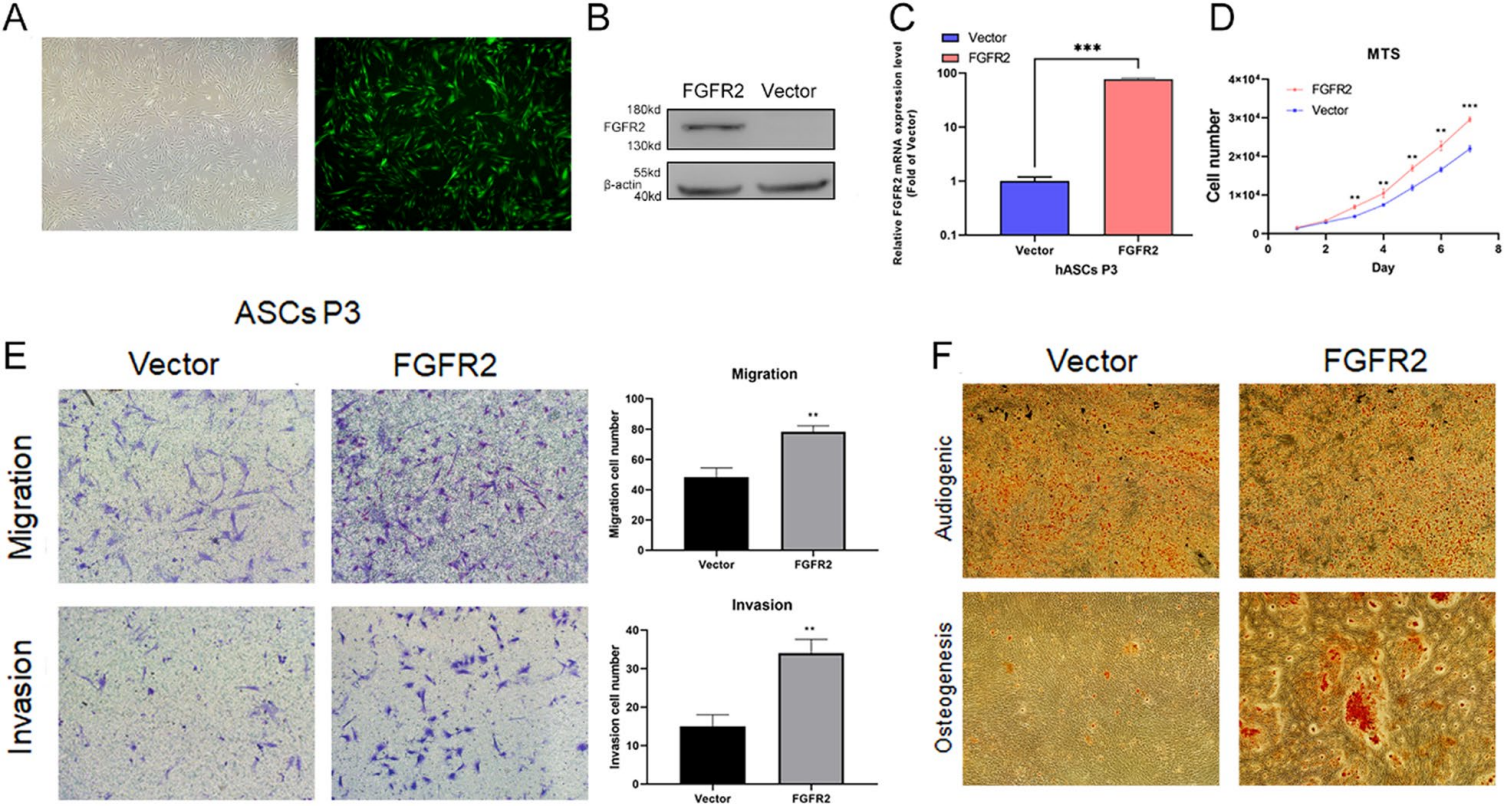
**A** ADSCs cells under white light and fluorescence; **B**, **C** Partial size of protein and mRNA expression levels of overexpressed FGFR2; **D**, **E** proliferation, migration and invasion ability of FGFR2 ctrl and OE ADSCs; **F** Osteogenic and adipogenic differentiation capacity of FGFR2 ctrl and OE ADSCs. “**”, < 0.01; “***”, < 0.001

**Fig. 5:**
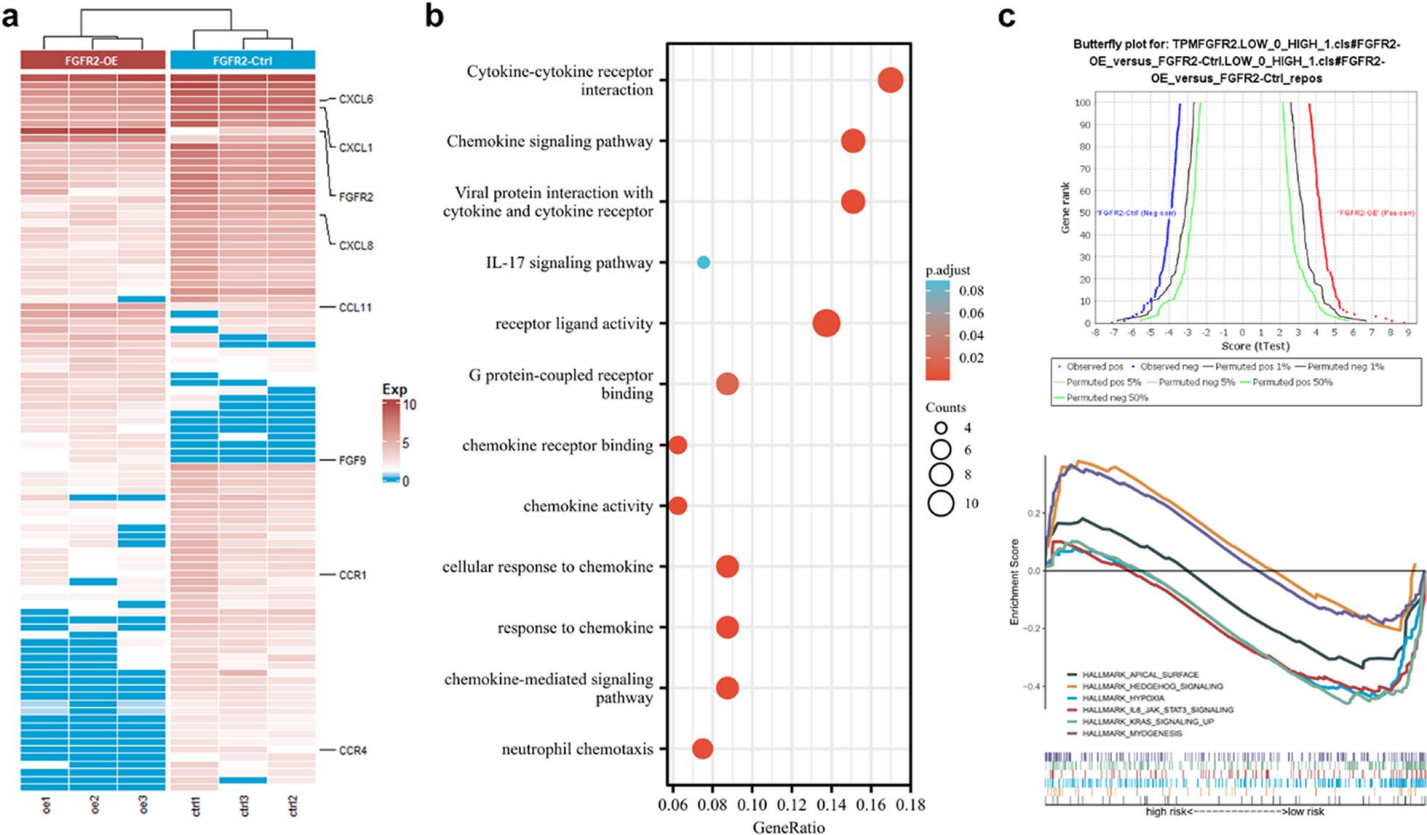
**A** Differential gene heatmap between two groups; **B** KEGG enrichment analyses of the differential genes; **C** GSEA enrichment analyses of the two groups

**Fig. 7:**
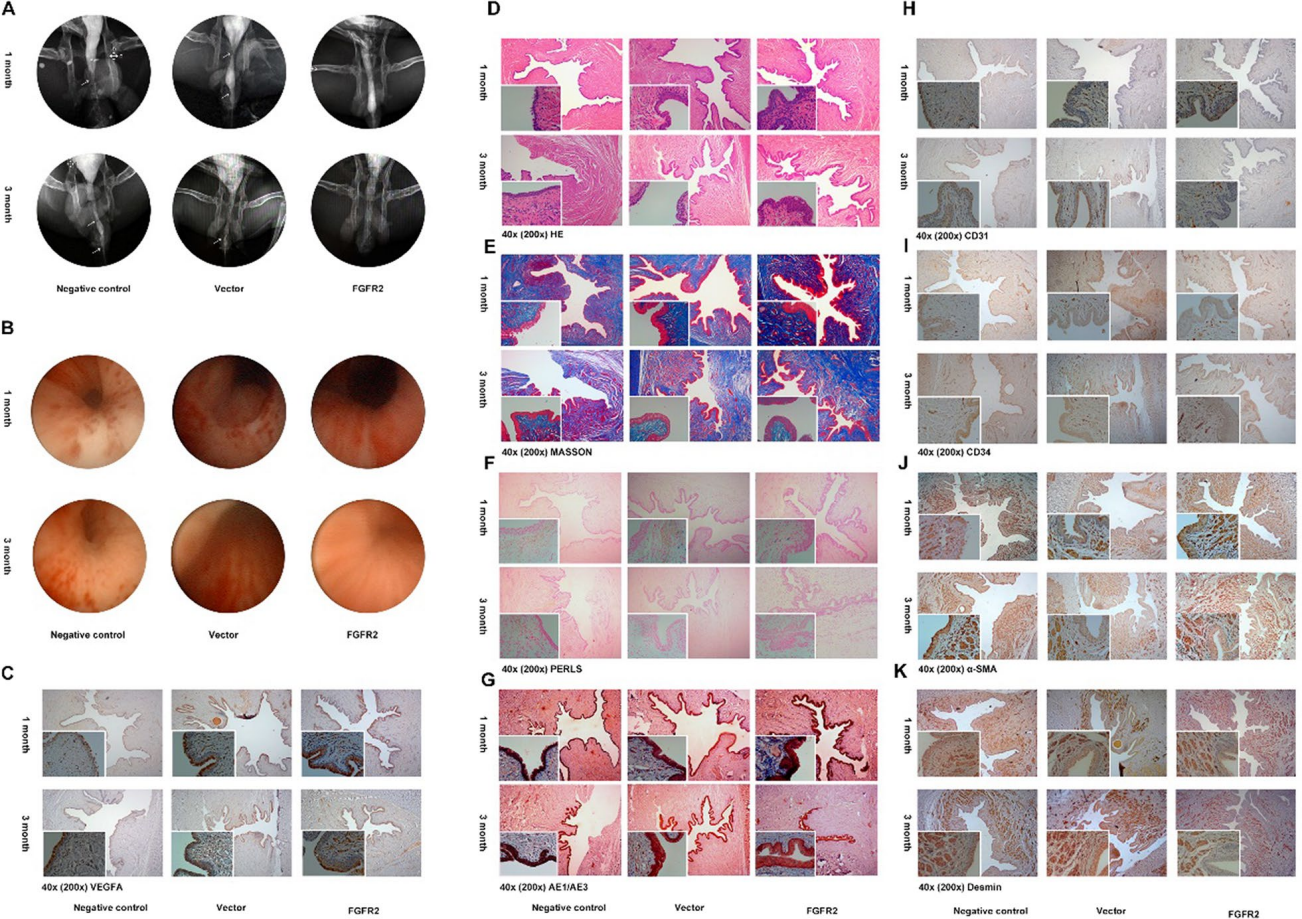
**A**, **B** Urography and urethroscopy of Negative controls, FGFR2 ctrl and FGFR2 OE groups at 1 month and 3 months after surgery; **C** VEGFA; **D** HE; **E** MASSON; **F** Prussian blue; **G** AE1/AE3; **H** CD31; **I** CD34; **J** alpha-SMA; **K** Desmin of Negative controls, FGFR2 ctrl and FGFR2 OE groups at 1 month and 3 months after surgery

In Fig. 2a, the image MBC/2um was duplicated from Fig. 3a, DMBC/2um in the team’s previous publication [2]. It has been replaced with parallel experiment of the microstructure of MBC in 2 μm. And all the images were added with a clearly standard ruler. The pictures only show the microstructure of the material and do not affect the experimental results. And in Fig. 1b, the authors corrected the wrong spelling of MBC to SBC.

In Fig. 4E, the magnification of Transwell in migration experiment was not consistent with that in invasion experiment, so the authors replaced the images with the same magnification in migration experiment. The statistics are based on the magnification statistics of Invasion, but there is a problem with the display picture, and the 3 repeated tests all show the similar results.

In Fig. 5C, the graphic drawing of GSEA enrichment analysis is wrong, but the analysis process and results are correct, which does not affect the conclusion, and the related graphics have been redrawn.

In Fig. 7C, magnified insert of 3 month/FGFR2 ctrl (vector) group was duplicated from 3 month/FGFR2 OE group. In Fig. 7J, the 1 month/FGFR2 negative group was corrected, magnified insert of 3 month/FGFR2 OE group was duplicated from 3 month/Negative control group, and the magnified insert of 3 month/FGFR2 vector group was incorrect. The 1 month/FGFR2 OE group and 3 month/FGFR2 ctrl (vector) group were misplaced and have been swapped. The authors provided the correct images to replace these erroneous ones.

In Additional file 1: Fig. S1A, due to the confusion of DMBC and SMBC in the related study, the 30-day degradation images of SMBC was duplicated from DMBC-30 day image in Additional file 1: Fig. S2 in the team’s previous publication [2]. So the authors corrected the 30-day degradation image of SMBC. And in Additional file 1: Fig. S1B, the BC/7 day image was duplicated from the SBC/100 um image in Fig. 2A. And for Additional file 1: Fig. S1B, although the relevant experimental material (e.g. SMBC/0 day) is a further representation of the pictures in Fig. 2, to eliminate the misunderstanding of repeated use of pictures, the authors replaced the relevant pictures in Additional file 1: Fig. S1B with the results of parallel experiments.

The authors apologize for the delay in finding the error in the above figures. The errors did not affect the results, and all relevant raw data were verified by the editorial team.

### Supplementary Information

**Additional file 1**.** Figure S1**. (A) In vitro degradation of BC and SMBC materials at 0 and 30 days. (B) Scanning electron microscopy microstructure of BC and SMBC materials at 0 and 7 days of in vitro degradation (100 µm)
