# Supplementary figures and images for: Clinical application of a double-modified sulfated bacterial cellulose scaffold material loaded with FGFR2-modified adipose-derived stem cells in urethral reconstruction
Source: Stem Cell Res Ther. 2022 Sep 6;13:463. doi: 10.1186/s13287-022-03164-9 (PMC9450280; doi:10.1186/s13287-022-03164-9)

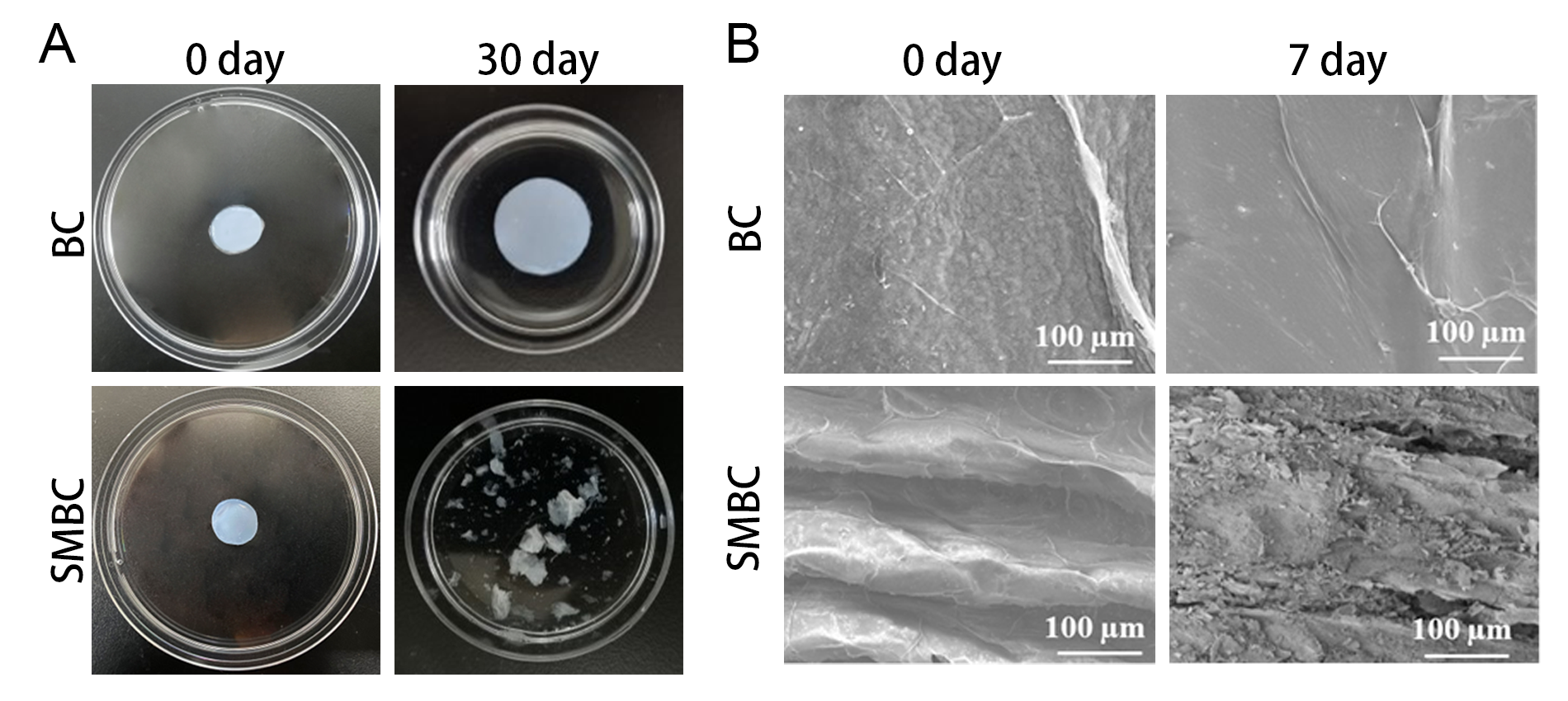

Supplement: Supplementary file 1 — Additional file 1. Figure S1. (A) In vitro degradation of BC and SMBC materials at 0 and 30 days. (B) Scanning electron microscopy microstructure of BC and SMBC materials at 0 and 7 days of in vitro degradation (100 µm) [file 13287_2022_3164_MOESM1_ESM.tif]
