# Supplementary material for: Correction: Clinical application of a double-modified sulfated bacterial cellulose scaffold material loaded with FGFR2-modified adipose-derived stem cells in urethral reconstruction
Source: Stem Cell Res Ther. 2024 Feb 27;15:53. doi: 10.1186/s13287-023-03541-y (PMC10898110; doi:10.1186/s13287-023-03541-y)
Supplement: Supplementary file 1 — Additional file 1. Figure S1. (A) In vitro degradation of BC and SMBC materials at 0 and 30 days. (B) Scanning electron microscopy microstructure of BC and SMBC materials at 0 and 7 days of in vitro degradation (100 µm) [file 13287_2023_3541_MOESM1_ESM.docx]

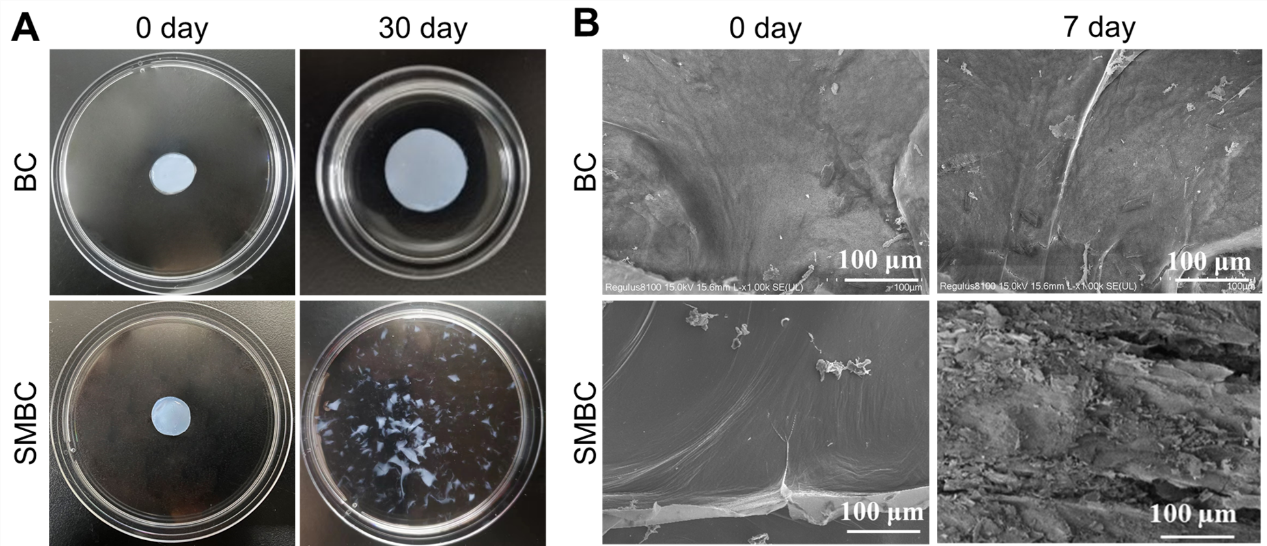


Fig S1. (A) In vitro degradation of BC and SMBC materials at 0 and 30 days. (B) Scanning electron microscopy microstructure of BC and SMBC materials at 0 and 7 days of in vitro degradation (100 µm)
